# Virus-induced Systemic Vasculitides: New Therapeutic Approaches

**DOI:** 10.1080/17402520400001744

**Published:** 2004

**Authors:** Loïc Guillevin

**Affiliations:** Department of Internal Medicine Hôpital Cochin, AP-HP Université René Descartes-Paris V Paris France

## Abstract

The best therapeutic strategy in virus-induced vasculitides should take into account the etiology of the disease and be adapted to the pathogenesis. The combination of antiviral treatments and plasma exchanges has been proven effective in polyarteritis nodosa (PAN). In human immunodeficiency virus (HIV)-related vasculitis this strategy is also effective and does not jeopardize, like cytotoxic agents, the outcome of AIDS. In vasculitis related to HCV-associated cryoglobulinemia, plasma exchanges improve the outcome but the poor effectiveness of antiviral drugs is not able to favor, usually, a definite recovery of the patients and relapses are frequent.


*Clinical & Developmental Immunology*, September/December 2004, Vol. 11 (3/4), pp. 227–231

Taylor & Francis healthsciences

### Virus-induced Systemic Vasculitides: New Therapeutic Approaches

LOÏC GUILLEVIN*

*Department of Internal Medicine, Hôpital Cochin, AP-HP, Université René Descartes-Paris V, 27, rue du Faubourg Saint-Jacques, Paris, France*

The best therapeutic strategy in virus-induced vasculitides should take into account the etiology of the disease and be adapted to the pathogenesis. The combination of antiviral treatments and plasma exchanges has been proven effective in polyarteritis nodosa (PAN). In human immunodeficiency virus (HIV)-related vasculitis this strategy is also effective and does not jeopardize, like cytotoxic agents, the outcome of AIDS. In vasculitis related to HCV-associated cryoglobulinemia, plasma exchanges improve the outcome but the poor effectiveness of antiviral drugs is not able to favor, usually, a definite recovery of the patients and relapses are frequent.

*Keywords*: Hepatitis B virus; Polyarteritis nodosa; Antiviral agents; Human immunodeficiency virus; Hepatitis C virus; Cryoglobulinemia

### INTRODUCTION

Viruses have been demonstrated to be the cause of several vasculitides. Two major vasculitides are the consequence of viral infection: hepatitis B virus (HBV) is responsible for classic polyarteritis nodosa (PAN) (Guillevin *et al.*, 1995) and hepatitis C virus (HCV) for mixed cryoglobulinemia (Ferri *et al.*, 2003). Other viruses can also be associated, albeit less frequently, to the occurrence of vasculitis, like human immunodeficiency virus (HIV) (Gherardi *et al.*, 1993), parvovirus B19 among others. The demonstration of a close relationship between viral infection and vasculitis has justifed a new therapeutic approach, avoiding prolonged administration of steroids and cytotoxic agents and based on the combination of antiviral agents and plasma exchange (PE). Herein, we focus on the management of virus-associated vasculitides.

### POLYARTERITIS NODOSA

A close relationship has been demonstrated between PAN and HBV infection (Trépo, 1972). Infections due to contaminated blood transfusions have now disappeared in developed countries, because of blood testing and donor selection, but that intravenous drug use is becoming the major cause of HBV-related PAN (Guillevin *et al.*, 1995). The development of vaccines against HBV and their administration to people at risk also explain the dramatic decrease of the number of new cases observed since 1989. During the 1970s, the prevalence of HBV infection in patients with classic PAN reached 50%. For the last 2 years, fewer than 10 patients a year have been identified throughout France (Guillevin *et al.*, 2004).

Some other viruses have been associated with the occurrence of PAN but could only explain the occurrence of a few cases per year. HCV does not seem to be a major etiological factor for PAN despite some descriptions (Cacoub *et al.*, 1992). GB virus-C, when sought in patients with PAN, has not been found to be an agent responsible for the disease (Servant *et al.*, 1998). When present, HCV was often observed in association with other viruses. Anecdotal parvovirus B19 infections have been described but a systematic survey of patients with PAN did not show a higher prevalence of parvovirus B19 in patients than the control population (Leruez *et al.*, 1994; Eden *et al.*, 2002).

The immunological process responsible for HBV-related PAN usually manifest less than 12 months after infection mainly in patients under 40 years of age. Hepatitis is rarely diagnosed, as it remains silent before the occurrence of PAN. Clinical manifestations are roughly the same as those commonly observed in PAN. The disease is acute. Seroconversion usually leads to recovery. The major sequelae are the consequence of vascular nephropathy and peripheral neuropathy but, even in patients who initially develop renal insufficiency, it is possible to cure PAN with little residual impairment of renal function. Rare cases of HIV-associated PAN (Gherardi *et al.*, 1993) have also been reported as well as Kawasaki disease (Johnson *et al.*, 2003). They do not

*Corresponding author. E-mail: loic.guillevin@cch.ap-hop-paris.fr

ISSN 1740-2522 print/ISSN 1740-2530 online © 2004 Taylor & Francis Ltd
DOI: 10.1080/17402520400001744

present specific characteristics and respond to the same therapeutic approach as PAN, due to other viral infections.

### Relapses

HBV-related PAN can be considered a self-limiting disease which tends not to recur once remission is induced. In our series (Guillevin *et al.*, 1995), only 6% of the patients relapsed. At present, it is still not possible to predict the subgroup of patients who will relapse, or the severity of the relapses. Due to the limited number of relapses, maintenance treatment is not necessary.

### Deaths

In all vasculitides, involvement of major organs can have lethal complications. A few patients die early from multivisceral involvement, often gastrointestinal, that cannot be controlled by treatment. In such cases, the course of the disease is generally characterized by fever, rapid weight loss, diffuse pain and involvement of one or several major organs involvement.

Conventional treatment with steroids and cyclophosphamide jeopardize the patient's outcome by allowing the virus to persist, thereby stimulating its replication and facilitating evolution towards chronic hepatitis and liver cirrhosis.

### Treatment of HBV-related PAN

For many years, HBV-related PAN was treated in the same way as non-virus-related PAN and patients received steroids, sometimes combined with cytotoxic agents, mainly cyclophosphamide. This treatment was often effective in short-term, but careful analysis of long-term results showed the occurrence of relapses and complications due to virus persistence, like chronic hepatitis or liver cirrhosis (McMahon *et al.*, 1989).

The rationale for combining PE and antiviral treatment was to obtain the following effects: initial corticosteroids to rapidly control the most severe life-threatening manifestations of PAN, which are common during the first weeks of the disease, and abrupt stoppage of corticosteroids to enhance immunological clearance of HBV-infected hepatocytes and favor HBe antigen (Ag) to anti-HBe antibody (Ab) seroconversion. PE can almost control the course of these PAN without the addition of steroids or cyclophosphamide.

In 1988, in conjunction with Christian Trépo we published (Trépo *et al.*, 1988; Guillevin *et al.*, 1988) the first results of a treatment combining antiviral treatment and PE, after a short-term steroid regimen. Steroids and/or immunosuppressants agents were abruptly discontinued to remove stimulants of virus replication, which was essential for antiviral treatment efficacy. An alternative therapy was also needed to reduce the mortality of PAN and to improve prognosis.

When this therapeutic strategy was first applied, the only available antiviral agent was vidarabine. After a 3-week course of vidarabine, administered after 1 week of steroids (1 mg/kg/d) and combined with PE, a full clinical recovery was obtained in three-quarters of the patients and HBeAb to anti-HBeAb seroconversion was observed in nearly half of the patients (Guillevin *et al.*, 1993). HBsAg to anti-HBsAb seroconversion was obtained only in 18% of the group. This latter low seroconversion rate was attributed to the limited efficacy of vidarabine and by the early integration of the virus genome into hepatocytes. Other antiviral agents like interferon-alpha (IFN-$\alpha$) (Guillevin *et al.*, 1994) or lamivudine (Guillevin *et al.*, 2004) should be preferred and gives better results than vidarabine. When prescribed, IFN-$\alpha$ dose is comparable to that prescribed for hepatitis B, i.e. 3 million units, 3 times a week. When no seroconversion is observed, after 4–6 months, the dose can be increased to 6 million units, 3 times a week. Tolerance of the treatment is in fact better than expected. The duration of the prescription does not exceed 6 months for the majority of them.

Lamivudine is an antiviral agent specifically designed for the treatment of HBV and HIV infections. In a small series of patients (Guillevin *et al.*, 2004), we perscribed 100 mg of lamivudine/day, in association with PE, after a few days steroids. Because lamivudine is eliminated by the kidney, the dose should be lower for patients with renal insufficiency and adapted to renal function. With this treatment, 60% of the patients seroconverted to anti-HBe and 90% recovered.

The severity of the disease in most patients requires therapy able to control immediately the severe or life-threatening manifestations of PAN. PE is able to rapidly clear the immune complexes (IC) responsible for the disease. This rapid intervention is the most appropriate to control the disease. In our protocol, steroids are also prescribed for a few days at the start of treatment to control as quickly as possible the clinical manifestations, while waiting for IFN-$\alpha$ efficacy to kick in. In our opinion, PE are not indicated because of their superiority to other medications but because they are, in combination with antiviral drugs, able to replace the deleterious therapies commonly used in virus-associated vasculitides with equivalent efficacy.

The optimal schedule is as follows: 4 sessions/week for 3 weeks, then 3 sessions/week for 2 to 3 weeks, followed by progressive lengthening of the intervals between sessions. One plasma volume (60 ml/kg) is usually exchanged using 4% albumin replacement fluid. The high number of PE can decrease the level of clotting factors and thereby lead to bleeding. Usually, the tolerance of PE is excellent, although some of our patients have experienced minor side effects.

One of the major advances obtained under the antiviral strategy was the very rapid cure of PAN, even in its most severe forms. It was sometimes possible to eliminate all signs of vasculitis more quickly, with some of our patients recovering within 3 weeks.

When antibodies directed against the virus are detected, plasmapheresis should be stopped to avoid the clearance of the newly synthesized antibodies. In a few cases, the Ab levels fluctuated, sometimes being present or absent. This Ag–Ab equilibrium can be very unstable and treatment should be continued. In such cases, it is more reliable to monitor virus activity by quantitative measurements of viral DNA.

### HCV CRYOGLOBULINEMIA

### Clinical Characteristics of HCV-related Cryoglobulinemia

Mixed cryoglobulinemia of type II and, more rarely, type III are the consequence of HCV infection in more than 80% of the patients (Agnello *et al.*, 1992). Cryoglobulinemia is asymptomatic in most patients but persists for decades and the disease duration might be a factor associated with the occurrence of clinical symptoms. When symptoms are present, the most frequent are purpura, peripheral neuropathy, glomerulonephritis, leg ulcers, arthritis and sicca syndrome. Relapses are common, and the disease is chronic even under treatment and when virus replication has stopped.

### Treatment

Only 10–20% of the patients with chronic hepatitis C achieved a sustained virological response. Polyethylene glycol (PEG)–IFN$\alpha$-2a increased the virological response in the recent studies where 69% of the patients had obtained a virological response at 48 weeks and a clinical recovery (Zeuzem *et al.*, 2000). A combination of IFN-$\alpha$ and ribavirin also increased the seroconversion rate in hepatitis C.

No treatment is able to cure the majority of the cases of mixed cryoglobulinemia definitively and an optimal therapeutic strategy has not yet been clearly defined. Steroids and immunosuppressive drugs are commonly used to treat severe forms, but they stimulate virus replication and can accelerate the development of chronic liver hepatitis and cirrhosis conditions, which facilitate the occurrence of liver cancer. As we did for HBV-related PAN, we devised a strategy associating antiviral drugs and PE for some patients (Cohen *et al.*, 1996). For asymptomatic patients, there is no argument to treat, and monitoring could be sufficient. In patients with moderate symptoms of cryoglobulinemia vasculitis (arthralgias, purpura, sensory peripheral neuropathy, for instance), IFN-$\alpha$ in combination with ribavirin should be tested.

#### *Interferon-alpha and other Antiviral Agents*

Treating cryoglobulinemia with IFN-$\alpha$ and ribavirin is based on the regimen prescribed for HCV hepatitis. Mixed cryoglobulinemia with clinical symptoms seems to be a disease that becomes manifest after decades-long chronic infection and, although there are no hard data to support a correlation between the time of infection and disease activity or between cryoglobulinemia level and disease severity, we hypothesize that it is more difficult to obtain virus clearance and disease remission during the chronic phase of cryoglobulinemia.

Clinical prospective trials using IFN-$\alpha$ alone (Cohen *et al.*, 1996), or in combination with PE and/or low-dose steroids (Cohen *et al.*, 1996; Rieu *et al.*, 2002) showed that the majority of patients improved clinically under treatment, that the replication rate decreased in the majority of the patients, but that a relapse occurred in 83% of the patients immediately after stopping treatment. In the prospective trials, patients were treated for only 6 months, which is obviously insufficient to obtain satisfactory results, but that was the recommended treatment duration for hepatitis at the time of the studies on cryoglobulinemia.

Combining two antiviral drugs, IFN-$\alpha$ and ribavirin is indicated. A correlation between virus activity and cryoglobulinemia also needs to be confirmed. Although majority of the patients seen for symptomatic cryoglobulinemia have virus-positive polymerase chain reactions (PCR), reflecting virus replication, a few of them remain serologically positive but become PCR negative, reflecting past contamination.

The indications of PE in HCV-related cryoglobulinemia are controversial. We recommend the combination of PE and antiviral drugs based on the effectiveness observed in many patients who failed to respond to other treatments and in severe clinical manifestations of cryoglobulinemia: chronic skin ulcers, glomerulonephritis, peripheral neuropathy. PE should not be prescribed systematically for every newly diagnosed case of cryoglobulinemia because the majority of patients present no or very few symptoms, and we do not know at present, whether or not treatment is indeed indicated in these pauci- or asymptomatic forms. Under PE, arteriolar ulcers regress quickly and complete healing can be obtained in a few weeks. PE should be tapered progressively to avoid a rebound phenomenon due to the increased production of cryoglobulins as a consequence of the stimulation of the B-cell clones responsible for their production. Some of our patients remain plasma exchange-dependent: clinical symptoms recur or worsen while tapering or after abrupt discontinuation of the sessions. New treatments could therefore be tried and anti-CD20 Ab are a promising effective drug that could improve the treatment of cryoglobulinemia (Sansonno *et al.*, 2003).

### HIV-ASSOCIATED VASCULITIS

### Clinical Characteristics

Vasculitides occurring during the course of HIV infection have been reported previously (Calabrese, 1989). Most of them involved skin, peripheral neuropathy or the central nervous system. The clinical spectrum and histological findings of HIV-related vasculitis vary widely (Gherardi *et al.*, 1993). Large-, medium- and small-sized arteries can be affected. Necrotizing arteritis, non-necrotizing arteritis, giant-cell arteritis, eosinophil arteritis and lymphomatoid granulomatosis have been reported (Gherardi *et al.*, 1993). According to Calabrese, the frequency is low (1%) (Calabrese, 1989), and most of the reported cases were identified at autopsy.

Some cases seem to be directly caused by opportunistic infections, such as *Pneumocystis carinii*, cytomegalovirus (CMV) or *Toxoplasma gondii*, non-opportunistic infectious agents or drug-induced hypersensitivity. In a few cases, HIV was thought to be the etiological agent because of the *in situ* localization of the virus and the absence of evidence suggesting other mechanisms. However, the etiology remains unknown in most cases.

The pathogenesis of virus-associated vasculitis is heterogeneous, but at least two general mechanisms have been incriminated: first, virus replication might induce direct injury of the vessel wall or vascular damage might be the result of an immune mechanism. These mechanisms may be cellular and/or humoral and include deposition of IC and/or their *in situ* formation.

### Treatment

The treatment of HIV-related vasculitis has the double objective to cure the vasculitis and to control the HIV infection. For this reason it seems of major importance not to prescribe steroids and cytotoxic agents. The first objective is to suppress HIV replication, which is more easily obtained with the combination of 2, 3 or 4 antiviral agents. Nucleotidic, non-nucleotidic inhibitors, and protease inhibitors are the most frequently used families of drugs. The treatment of vasculitis is not easy to establish, because the pathogenetic mechanisms of the disease are not completely understood. Based on the presence of CIC, we have proposed (Gisselbrecht *et al.*, 1997), as for other virus-associated vasculitides, to treat the patients with PE, using the same scheme as that to HBV-related PAN. In our clinical experience, this association was successful and the first 8 patients we treated improved and remissions were obtained. In some patients who presented with co-infections, i.e. HCV and HIV or HBV and HIV, this strategy was also effective. Some patients with cryoglobulinemia responded very quickly to the regimen.

HIV-related vasculitides appear to be a one-shot disease and do not recur. A short treatment, 1 to 3 months, is usually sufficient to cure the disease.

### MISCELLANEOUS

Other viruses have also be described in association with vasculitis: parvovirus, Epstein Barr virus etc. It is sometimes difficult to establish a close relationship between viral infection and vasculitis. Recently, we successfully treated a patient who had developed PAN after parvovirus infection with intravenous immunoglobulins alone; the patient recovered quickly (Viguier *et al.*, 2001).

### *References*

Agnello, V., Chung, R.T. and Kaplan, L.M. (1992) "A role for hepatitis C virus infection in type II cryoglobulinemia", *N. Engl. J. Med.* **327**(21), 1490–1495.

Cacoub, P., Lunel-Fabiani, F. and Du, L.T. (1992) "Polyarteritis nodosa and hepatitis C virus infection", *Ann. Intern. Med.* **116**(7), 605–606.

Calabrese, L. (1989) "The rheumatic manifestations of infection with the human imunodeficiency virus", *Semin. Arthritis Rheum.* **18**, 225–229.

Cohen, P., Nguyen, Q.T., Deny, P., Ferriere, F., Roulot, D., Lortholary, O., *et al.* (1996) "Treatment of mixed cryoglobulinemia with recombinant interferon alpha and adjuvant therapies. A prospective study on 20 patients", *Ann. Med. Interne* **147**(2), 81–86.

Eden, A., Mahr, A., Servant, A., Radjef, N., Amard, S., Mouthon, L., *et al.* (2002) "Serologic and molecular parvovirus B19 analyses in ANCA-associated vasculitides: a case-control study", *Clevel. Clin. J. Med.* **69**(Suppl. 2), 156–157, (abstract).

Ferri, C., Giuggioli, D., Cazzato, M., Sebastiani, M., Mascia, M.T. and Zignego, A.L. (2003) "HCV-related cryoglobulinemic vasculitis: an update on its etiopathogenesis and therapeutic strategies", *Clin. Exp. Rheumatol* **21**(6 Suppl. 32), S78–S84.

Gherardi, R., Belec, L., Mhiri, C., Gray, F., Lescs, M.C., Sobel, A., *et al.* (1993) "The spectrum of vasculitis in human immunodeficiency virus-infected patients. A clinicopathologic evaluation", *Arthritis Rheum.* **36**(8), 1164–1174.

Gisselbrecht, M., Cohen, P., Lortholary, O., Jarrousse, B., Gayraud, M., Ghérardi, R., *et al.* (1997) "HIV-related vasculitis: clinical presentation and therapeutic approach on six patients [letter]", *Aids* **11**(1), 121–123.

Guillevin, L., Merrouche, Y., Gayraud, M., Jarrousse, B., Royer, I., Leon, A., *et al.* (1988) "Périartérite noueuse due au virus de l'hépatite B. Détermination d'une nouvelle stratégie thérapeutique chez 13 patients", *Presse Med.* **17**(30), 1522–1526.

Guillevin, L., Lhote, F., Leon, A., Fauvelle, F., Vivitski, L. and Trepo, C. (1993) "Treatment of polyarteritis nodosa related to hepatitis B virus with short term steroid therapy associated with antiviral agents and plasma exchanges. A prospective trial in 33 patients", *J. Rheumatol.* **20**(2), 289–298.

Guillevin, L., Lhote, F., Sauvaget, F., Deblois, P., Rossi, F., Levallois, D., *et al.* (1994) "Treatment of polyarteritis nodosa related to hepatitis B virus with interferon-alpha and plasma exchanges", *Ann. Rheum. Dis.* **53**(5), 334–337.

Guillevin, L., Lhote, F., Cohen, P., Sauvaget, F., Jarrousse, B., Lortholary, O., *et al.* (1995) "Polyarteritis nodosa related to hepatitis B virus. A prospective study with long-term observation of 41 patients", *Medicine (Baltim)* **74**(5), 238–253.

Guillevin, L., Mahr, A., Cohen, P., Larroche, C., Queyrel, V., Loustaud-Ratti, V., *et al.* (2004) "Short-term corticosteroids then lamivudine and plasma exchanges to treat hepatitis B virus-related polyarteritis nodosa", *Arthritis Rheum.* **51**(3), 482–487.

Johnson, R.M., Barbarini, G. and Barbaro, G. (2003) "Kawasaki-like syndromes and other vasculitic syndromes in HIV-infected patients", *Aids* **17**(Suppl. 1), S77–S82.

Leruez, V.M., Lauge, A., Morinet, F., Guillevin, L. and Deny, P. (1994) "Polyarteritis nodosa and parvovirus B19 [letter]", *Lancet* **344**(8917), 263–264.

McMahon, B.J., Heyward, W.L., Templin, D.W., Clement, D. and Lanier, A.P. (1989) "Hepatitis B-associated polyarteritis nodosa in Alaskan Eskimos: clinical and epidemiologic features and long-term follow-up", *Hepatology* **9**(1), 97–101.

Rieu, V., Cohen, P., André, M., Mouthon, L.P.G., Jarrousse, B., *et al.* (2002) "Characteristics and outcome of 49 patients with symptomatic cryoglobulinaemia", *Rheumatology (Oxf)* **41**, 290–300.

Sansonno, D., De Re, V., Lauletta, G., Tucci, F.A., Boiocchi, M. and Dammacco, F. (2003) "Monoclonal antibody treatment of mixed cryoglobulinemia resistant to interferon alpha with an anti-CD20", *Blood* **101**(10), 3818–3826.

Servant, A., Bogard, M., Delaugerre, C., Cohen, P., Dény, P. and Guillevin, L. (1998) "GB virus C in systemic medium- and small-vessel necrotizing vasculitides", *Br. J. Rheumatol* **37**, 1292–1294.

Trépo, C. (1972) "Hepatite virale B et périartérite noueuse", *Nouv. Presse Med.* **1**(28), 1879–1881.

Trépo, C., Ouzan, D., Delmont, J. and Tremisi, J. (1988) "Supériorité d'un nouveau traitement étiopathogénique guérissant la périartérte noueuse due au virus de l'hépatite B par la combinaison d'une brève corticothérapie, de vidarabine et d'échanges plasmatiques", *Presse Med.* **17**(30), 1527–1531.

Viguier, M., Guillevin, L. and Laroche, L. (2001) "Treatment of parvovirus B19-associated polyarteritis nodosa with intravenous immune globulin", *N. Engl. J. Med.* **344**(19), 1481–1482.

Zeuzem, S., Feinman, S., Rasenack, J., *et al.* (2000) "Peginterferon alpha-2a in patients with chronic hepatitis C", *N. Engl. J. Med.* **343**, 1666–1672.

